# Voxel based comparison and texture analysis of ^18^F-FDG and ^18^F-FMISO PET of patients with head-and-neck cancer

**DOI:** 10.1371/journal.pone.0213111

**Published:** 2019-02-28

**Authors:** Markus Kroenke, Kenji Hirata, Andrei Gafita, Shiro Watanabe, Shozo Okamoto, Keiichi Magota, Tohru Shiga, Yuji Kuge, Nagara Tamaki

**Affiliations:** 1 Department of Nuclear Medicine, Klinikum rechts der Isar, Technical University Munich, Munich, Germany; 2 Department of Nuclear Medicine, Graduate School of Medicine of Hokkaido University, Sapporo, Japan; 3 Central Institute of Isotope Science, of Hokkaido University, Sapporo, Japan; Technische Universitat Munchen, GERMANY

## Abstract

**Background:**

Hypoxia can induce radiation resistance and is an independent prognostic marker for outcome in head and neck cancer. As ^18^F-FMISO (FMISO), a hypoxia tracer for PET, is far less common than ^18^F-FDG (FDG) and two separate PET scans result in doubled cost and radiation exposure to the patient, we aimed to predict hypoxia from FDG PET with new techniques of voxel based analysis and texture analysis.

**Methods:**

Thirty-eight patients with head-and-neck cancer underwent consecutive FDG and FMISO PET scans before any treatment. ROIs enclosing the primary cancer were compared in a voxel-by-voxel manner between FDG and FMISO PET. Tumour hypoxia was defined as the volume with a tumour-to-muscle ratio (TMR) > 1.25 in the FMISO PET and hypermetabolic volume was defined as >50% SUVmax in the FDG PET. The concordance rate was defined as percentage of voxels within the tumour which were both hypermetabolic and hypoxic. 38 different texture analysis (TA) parameters were computed based on the ROIs and correlated with presence of hypoxia.

**Results:**

Within the hypoxic tumour regions, the FDG uptake was twice as high as in the non-hypoxic tumour regions (SUVmean 10.9 vs. 5.4; p<0.001). A moderate correlation between FDG and FMISO uptake was found by a voxel-by-voxel comparison (r = 0.664 p<0.001). The average concordance rate was 25% (± 22%). Entropy was the TA parameter showing the highest correlation with hypoxia (r = 0.524 p<0.001).

**Conclusion:**

FDG uptake was higher in hypoxic tumour regions than in non-hypoxic regions as expected by tumour biology. A moderate correlation between FDG and FMISO PET was found by voxel-based analysis. TA yielded similar results in FDG and FMISO PET. However, it may not be possible to predict tumour hypoxia even with the help of texture analysis.

## Introduction

Hypoxic regions in tumours are known to be more radiation-resistant than normoxic tissues and hypoxia is an independent prognostic marker for patient outcome [1–5]. Most head-and-neck cancers have hypoxic regions, which vanish in the course of radiation therapy [6]. The current gold standard to estimate hypoxic tissue is measuring the pO2 using the Eppendorf pO2 electrode [7], which not only is an invasive method but also can alter the local oxygen concentration. Fluorine-18-labeled fluoromisonidazole (FMISO) positron emission tomography (PET) is a known technique that allows visualisation of hypoxic areas with high reproducibility [8–10]. New therapeutic strategies have been proposed to treat hypoxic tumour areas, such as dose escalation and de-escalation of radiation therapy or dose painting (increased dosage in hypoxic regions of the tumour) [11–13], as well as inhibitors for hypoxia-inducible factor (HIF) [14]. Fluorine-18-labeled fluorodeoxyglucose (FDG) is a common imaging agent used in the clinical routine to detect malignant tumours and to evaluate its degree of aggressiveness. Significant correlation was found between pO_2_ and FMISO-PET uptake in head-and-neck cancer, and a moderate correlation of FDG and FMISO uptake [15], while others reported that the FDG PET could not predict hypoxic areas [16]. On the other side, no correlation between FDG- and FMISO-PET was found in lung cancer [17].

So far, most reports focused on assessing the SUVmax of the tumour, while data from voxel-based analyses, such as metabolic tumour volume (MTV) and total lesion glycolysis (TLG) are lacking [18]. In addition, texture analysis is increasingly used to quantify image heterogeneity in PET [18–22]. In xenograft tumours of head-and-neck cancer cells, increasing metabolic heterogeneity was found to reflect tumour hypoxia [23]. We hypothesized that hypoxia areas may develop many small necrotic foci and thus metabolic heterogeneity might reflect hypoxia. Therefore, we assessed the potential of using texture analysis of the tumour based on FDG PET images to predict areas with high uptake in FMISO PET.

The aim of this study was to evaluate the correlation between FDG- and FMISO-PET imaging in head and neck tumours using with various techniques of image analysis and to get a better understanding of tumour hypoxia on a macroscopic point of view and to test if FDG can predict hypoxia using new computational tools.

## Materials and methods

### Patients

A total of 38 patients (7 female and 31 male) with untreated head and neck cancer were prospectively enrolled from February 2009 to February 2015 in this study. Signed informed consent was obtained from all patients and the study was approved by the Institutional Review Board of Hokkaido University (No 10–0094). Additional information regarding the patients can be found in S1 Table.

### Image acquisition

For FMISO-PET, 400 MBq of FMISO was intravenously injected for each patient without fasting. Emission scanning started 4 hours after injection. Head to upper thorax was scanned for 10 minutes. The relatively long interval allowed sufficient clearance of the tracer from the blood pool. For FDG-PET, 4.5MBq/kg of ^18^F-FDG was injected after at least 6 hours of fasting. One hour after injection, the head to upper thorax was scanned for 10 minutes. Blood glucose levels of all patients were measured before FDG injection and were confirmed to be below 140 mg/dl. All PET scans were performed on a TruePoint Biograph 64 PET-CT scanner (Siemens Japan, Tokyo, Japan) with TrueV option. The transaxial and axial fields of view were 68.4 cm and 21.6 cm, respectively. An integrated non-contrast-enhanced CT (NCE-CT) was conducted for attenuation correction and anatomical registration purposes. The images were reconstructed with the iterative TrueX reconstruction method, which included point spread function correction [24]. The full width at half maximum after reconstruction was 8 mm. The voxel size was 3.0 x 3.0 x 3.0 mm^3^. Each patient underwent FMISO PET and FDG PET within one week. The enhanced magnetic resonance (MR) scans were performed within one month with the PET scans. The MR scan included axial T2 weighted fat saturated images and T1 weighted fat saturated images with contrast enhancement sequences.

### Image analysis

Three analyses were performed: 1) comparison of FMISO- and FDG PET-derived tumour burden parameters, 2) voxel-wise image comparison of PET images and 3) texture analyses.

Values are shown in mean ± 1 standard deviation (SD), p<0.05 was considered statistically significant. Parametric data was assessed via Pearson’s correlation coefficient and Student’s t-test while non-parametric data was tested via Spearman’s rank correlation coefficient. TA parameters were analysed via correlation matrices. The statistical analysis was performed using Microsoft Excel 365 (2017) and IBM SPSS 22 (2015).

#### Tumour burden parameters

FDG PET-derived parameters (MTV, HV, SUVmean, SUVmax), FMISO PET-derived (HV, SUVmean, SUVmax) and the presence of necrosis area in MR images were compared in-between.

#### Voxel-wise image comparison

Firstly, FDG- and FMISO-PET datasets were automatically coregistered using an IntelliSpace Portal version 5 (2012 Philips, Amsterdam, Netherlands) workstation followed by minimal manual corrections when necessary. The precision of coregistration was visually confirmed by two experienced nuclear medicine physicians.

Regions of interest (ROI) were manually created in the FDG PET on each slice showing the tumour to roughly enclose the entire tumour. In case lymph nodes were not reliably separable from the primary tumour, the lymph nodes were included in the ROI. Otherwise, all the other metastatic lesions were excluded from the ROI. The ROIs were then transferred to the FMISO PET. Based on the manual ROI the metabolic tumour volume (MTV) was calculated using a fixed threshold of SUV>2.5 [25,26]. A hypermetabolic volume (HMV) was defined as the volume having a higher uptake than 50% of the SUVmax (FDG) of the lesion. All voxels above this threshold were retrieved from both FDG and FMISO PET datasets. The FMISO uptake was normalised by using the TMR as a high reproducibility was shown before [8,27]. A voxel with a TMR > 1.25 in the FMISO PET was defined as hypoxic. Hypoxic volume (HV) was calculated by counting all hypoxic voxels.

Two different concordance rates were defined as follows. The concordance rates were derived for every patient individually using Microsoft Excel 365 (2017). The concordance rate c was defined in Eq 1

$$\frac{A\cap B}{A\cup B}\;=\;c$$ (1)

A: = hypermetabolic volume

B: = hypoxic volume

The whole concordance rate c_w_ is defined in Eq 2

$$\frac{A\cap B}{A\cup B}\;=\;c_{}$$ (2)

C: = Tumor volume without hypermetabolic tumour volume

#### Texture analysis

The texture analysis was conducted using an in-house developed tool in R 3.4.0 upon the before described ROI. We firstly calculated first-order statistics, where voxel location was not considered, including Standard Deviation, Skewness, Kurtosis, EntropyHist, and EnergyHist. Secondly, 4 matrices were generated for higher-order statistics consisting of Gray-level cooccurence matrix (GLCM), Gray-level run length matrix (GLRLM), Neighborhood gray-level different matrix (NGLDM), and Gray-level zone size matrix (GLZSM), as described [19]. GLCM was calculated from 13 different directions in 3-D space and generated: Homogeneity, Energy, Correlation, Contrast, Entropy and Dissimilarity. GLRLM was calculated from 13 different directions and generated: SRE, LRE, HGRE, LGRE, SRLGE, SRHGE, LRLGE, LRHGE, GLNUr, RLNU and RP. NGLDM, in which the 26 nearest neighbours in 3-D space were involved, generated Coarseness and Contrast. GLZSM did not require calculations in several directions and generated: SZE, LZE, LGZE, HGZE, SZLGE, SZHGE LZLGE, LZHGE, GLNUz, ZLNU and ZP. Gray-level resampling step was fixed as 64 in the current study. Detailed information about above mentioned radiomics parameters are given in Table 1 and in S1 Dataset.

**Table 1:** TA paremeter abbreviations.

SRE	short-run emphasis
LRE	long-run emphasis
LGRE	low grey-level run emphasis
HGRE	high grey-level run emphasis
SRLGE	short-run low grey-level emphasis
SRHGE	short-run high grey-level emphasis
LRLGE	long-run low grey-level emphasis
LRHGE	long-run high grey-level emphasis
GLNUr	grey-level non-uniformity for run
RLNU	run-length non-uniformity
RP	run percentage
SZE	short-zone emphasis
LZE	long-zone emphasis
LGZE	low grey-level zone emphasis
HGZE	high grey-level zone emphasis
SZLGE	short-zone low grey-level emphasis
SZHGE	short-zone high grey-level emphasis
LZLGE	long-zone low grey-level emphasis
LZHGE	long-zone high grey-level emphasis
GLNUz	grey-level non-uniformity for zone
ZLNU	zone length non-uniformity
ZP	zone percentage

The raw voxel data were exported as text files. Image noise in PET imaging in volumes smaller than 1 ml is a well-known phenomenon, therefore we created an artificial threshold of a hypoxic volume of at least 1 ml. For MR images, the primary tumours were assessed by two experienced physicians per three-point scale (no necrosis, possible necrosis and necrosis). Discrepancy between 2 physicians was resolved by discussion.

Univariate logistic regressions were performed using hypoxia state as dependent variable and PET-based parameters (SUVmax, SUVmean, histologic grade (WHO classification) and TAs) as independent variables. Only TA that showed a high correlation (>0.5) between FDG-PET and FMISO-PET were included in the analyses. In a second step logistic regressions were used to distinguish non-hypoxic tumours and small HV tumours (< 1 ml) from hypoxic tumours with a HV (> 1 ml). This threshold was artificially created as only 5 patients had no hypoxic voxels at all and a few patients had very few hypoxic voxels, probably due to image noise.

## Results

### Patients

38 patients were included in this study. Mean (SD) age was 59 (range: 38 to 80 years, SD 10 years). Thirty-three patients had a nasopharyngeal carcinoma, 4 an oropharyngeal carcinoma and 1 a laryngeal carcinoma. Most of the patients (79%) suffered from advanced tumour stages (III or IV): only three patients were in UICC tumour stage I, five in stage II, nineteen in stage III and eleven in stage IV.

### Imaging

Patients were injected with 414.5 ± 27 MBq ^18^F-FMISO and 358.6 ± 54 MBq ^18^F-FDG, respectively. The scanning time started 258 ± 23 min and 86 ± 15 min after injection for FMISO PET and FDG PET, respectively. Time between the two PET scans was in average 4.3 ± 5 days (range 1–23 days).

SUVmax and SUVmean were 17.2 ± 7 (range 5.4–33.8) and 6.1 ± 2 (3.4–10.4) for FDG-PET and 2.5 ± 0.9 (1.21–5.0) and 1.3 ± 0.3 (1.0–3.0) for FMISO-PET. The TMRmax was 1.8 ± 0.5 (1.0–3.0) and the TMRmean 0.9 ± 0.1 (0.7–1.2). The MTV was 39.6 ± 26 ml (2.1–102.4 ml) whereas the HMV in the FDG PET was 10.4 ± 9 ml (0.7–33.6 ml). The HV was 4.8 ± 6.8 ml (0–32.1 ml), and when excluding the 5 non-hypoxic tumours, HV was 5.6 ± 7 ml (0.1–32.1 ml).

### Tumor burden parameters comparison

**Fig 1:**
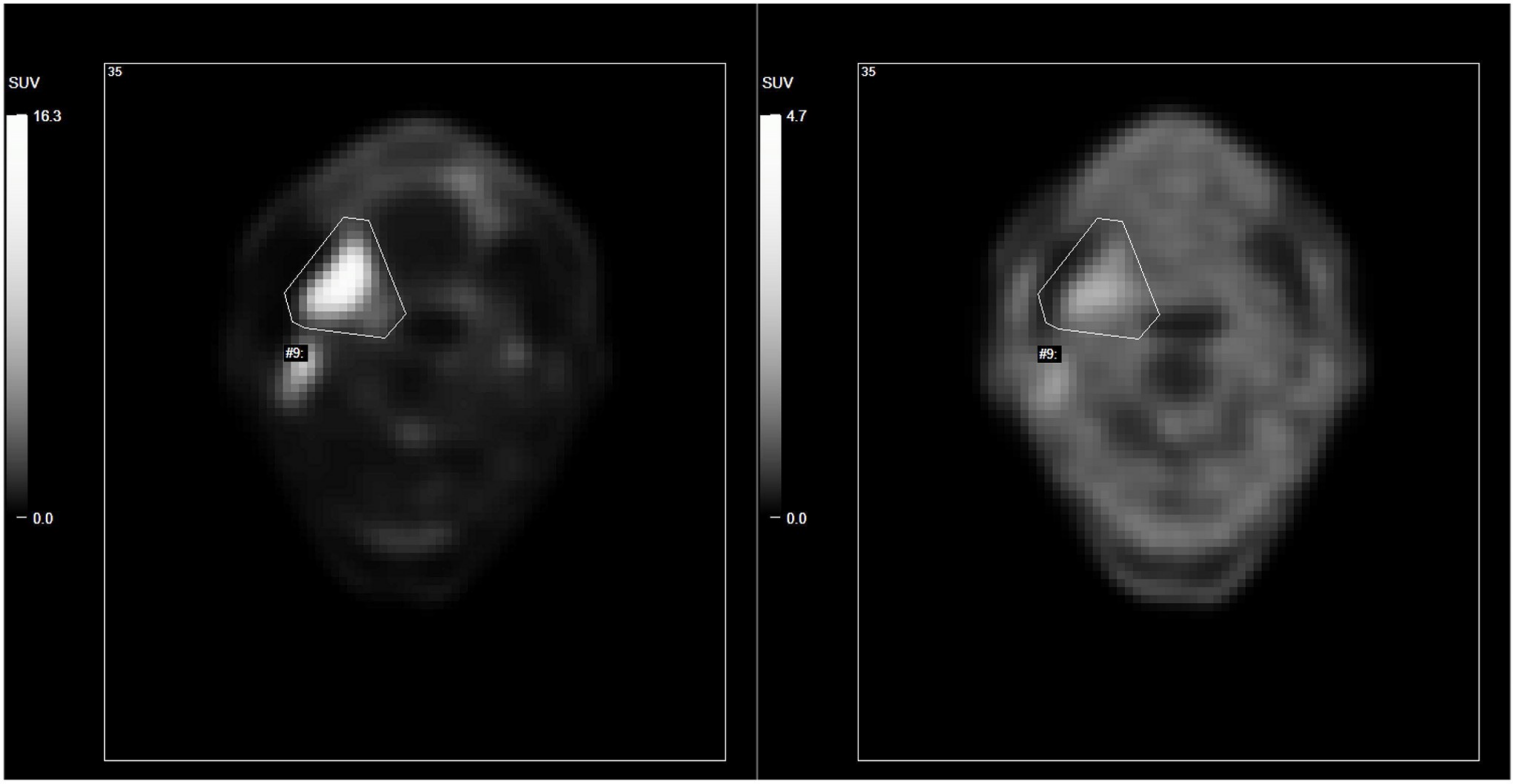
Side by side comparison of FDG (left) and FMISO (right) PET image of a representative patient with a SCC on the right side surrounded by the manual ROI. Note: different contrast ratio selected for each PET image.

A moderate significant correlation was noticed between SUVmax obtained in FDG-PET and FMISO-PET (r = 0.44, p<0.001), and between TLG and SUVmax in FMISO-PET (r = 0.44, p<0.001). Fig 1 shows a representative example of FDG- and FMISO-PET images with the manual ROI. In FDG-PET imaging, SUVmean from hypoxic areas was significantly higher than in non-hypoxic areas (10.9 vs 5.4, p< 0.001). Seven primary tumours showed necrosis in the MRI, three possible necrosis and 28 exhibited no necrosis. There were no significant differences of SUVmax (FMISO) between the groups (no necrosis vs. possible necrosis vs. necrosis).

### Voxel-wise image correlation

The average Pearson’s correlation coefficient of the voxel based correlation was 0.68 ± 0.17 (p<0.001) based on the FDG PET SUV>2.5 ROI, which was slightly higher than based on the manual ROI (r = 0.61 ± 0.20, p<0,001). Seventeen of 38 (45%) patients showed high (r>0.7) and 18 (48%) intermediate (0.7>r>0.4) correlations.

**Fig 2:**
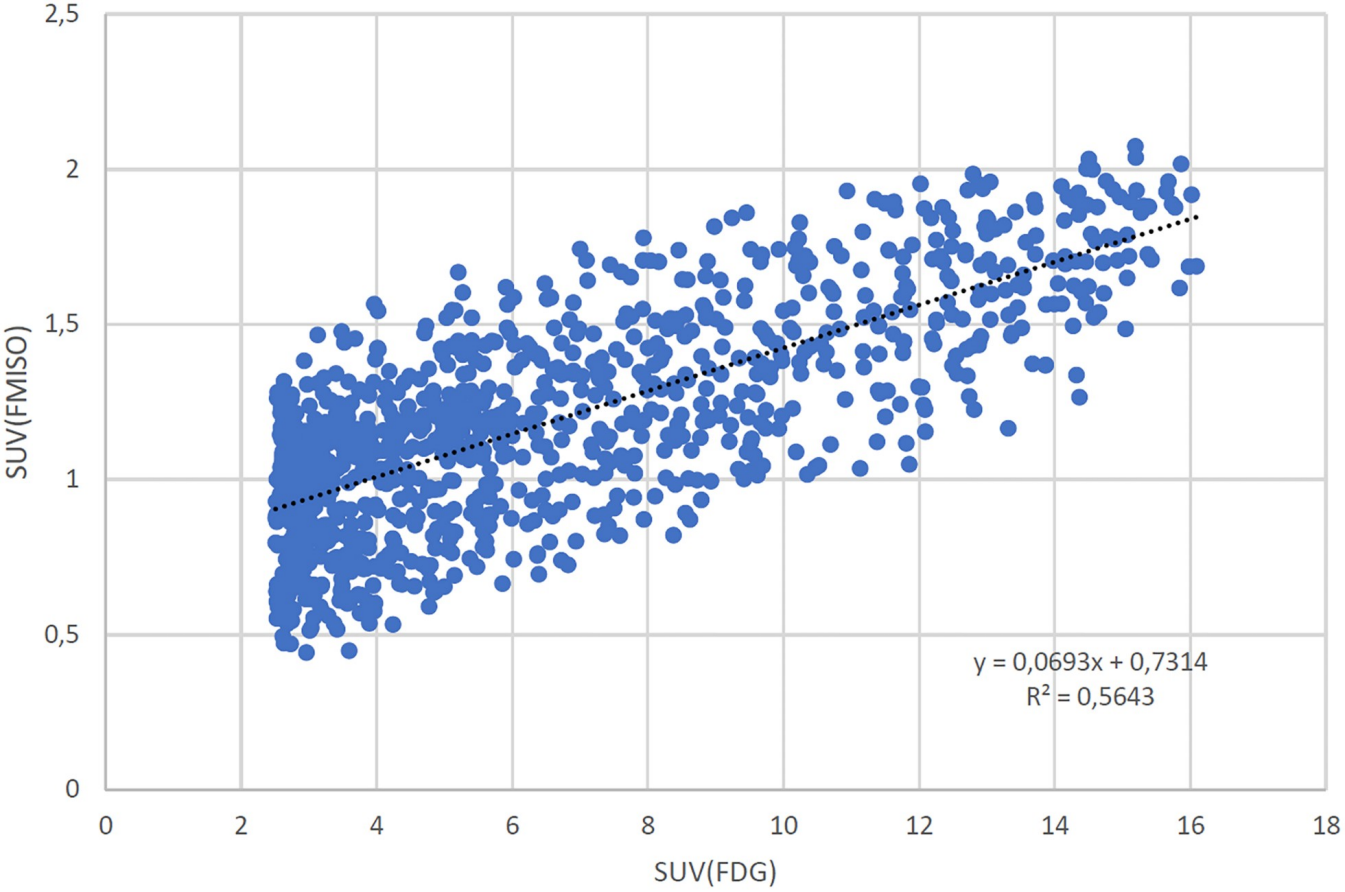
Corresponding to Fig 1 the scatter plot of SUV (FDG) and SUV (FMISO) voxel vice.

Fig 2 shows corresponding to Fig 1 the correlation of all voxels included in the ROI (SUV>2.5) of one patient.

No correlation was found between the concordance rate and MTV (r = 0.005, p<0,001) and HMV (r<0.001, p<0,001), although it can be noted that FMISO negative tumours were all smaller than 7 ml in MTV. The average concordance rate c was 25 ± 22%, whereas it was 29 ± 20% when the 5 FMISO negative tumours were not considered. The whole concordance rate c_w_ was 77 ± 15%.

SUVmax(FMISO) and the (positive) concordance rate were independent from tumour stage (r_s_ = 0.118 and 0.057, respectively). MTV and SUVmax(FMISO) were correlated weakly but significantly (r = 0.312, p = 0.028) whereas HMV and SUVmax(FMISO) were not correlated (r = -0.011, p = 0.474).

Neither FDG nor FMISO uptake (mean and max) did correlate with the tumour stage or the WHO histopathology grading.

### Texture analysis

High correlation/Linear dependence between each pair of the FDG texture parameter (40 x 40 table): 8 groups with r>0.8 p<0.001 were found. 35 of 40 parameters (87.5%) were in similar groups as found by Orlhac et al. 2014 [19].

**Fig 3:**
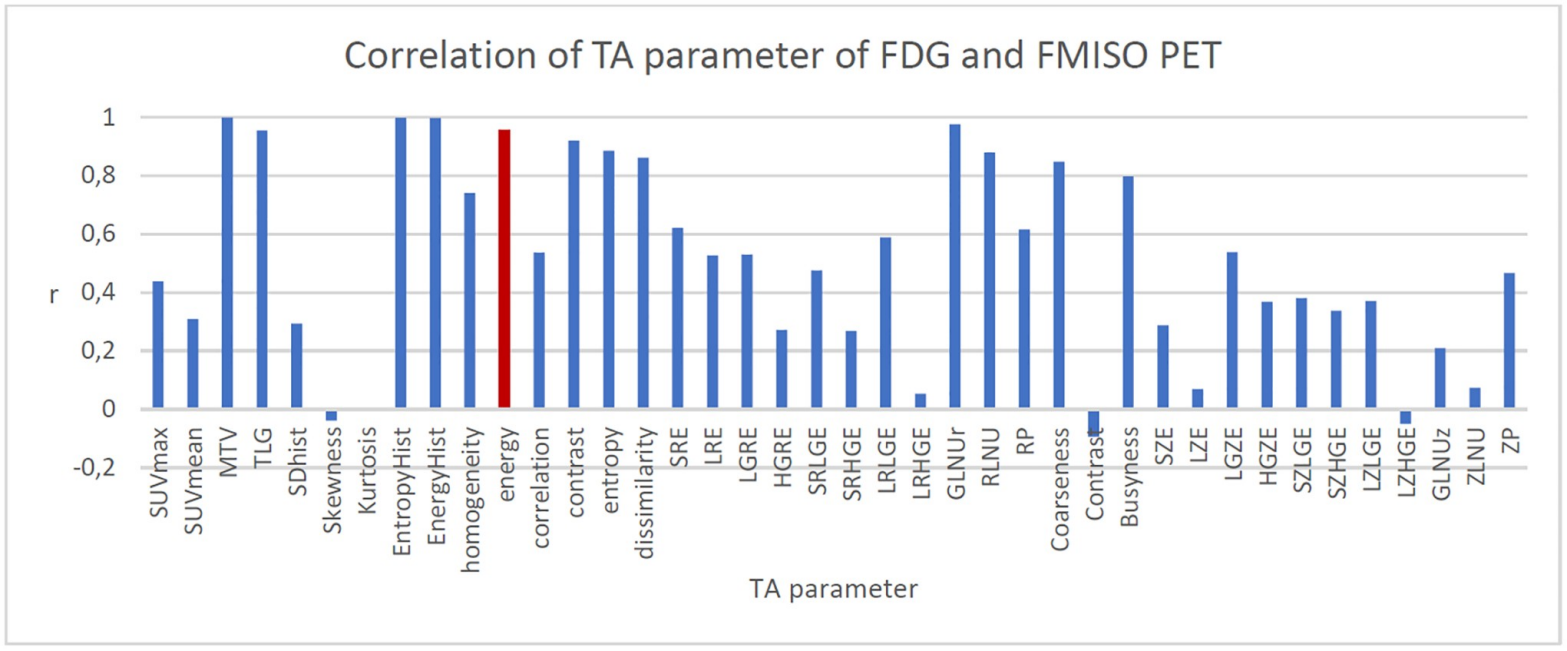
All correlations are statistical significant (p<0.05) except Engergy (red bar).

Strong correlation (r>0.8) between TA (FDG) and TA (FMISO) parameters was only found in groups 2 and 4 (Table 2). Other TA parameters were less correlated with each other (r<0.5). Entropy showed the highest correlation with presence of hypoxia (r = 0.524) and lower than r = 0.361 with TMR, HV, hypoxia based on volumes >1 ml (binary) and >2.24 ml (median hypoxic volume) measured in FMISO PET. All other parameters showed correlation mostly well below r = 0.4 with Hypoxia, TMR and HV and hypoxic volume. Fig 3 shows that there are only moderate correlations between the SUVmax(FMISO) and the TA parameter of the FDG-PETs. The highest correlations are found for skewness 0.4 and strongest negative correlations around -0.4 for contrast and dissimilarity.

**Table 2:** TA parameter grouped according to highest correlation (>0.8 p<0.05). The TA parameter “Correlation” did not correlate with any other parameter.

Group	TA Parameter
1	SUVmax, SUVmean, Sdhist (G), HGRE (u), SRHGE, LRHGE, Contrast, HGZE, SZHGE
2	MTV, TLG, GLNUr, RLNU, Busyness, GLNUz, ZLNU
3	Skewness, Kurtosis
4	EntropyHist, EnergyHist, Homogenity, Energy, Contrast, Entropy, Dissimilarity, Coarseness
5	SRE, LRE, RP, ZP, SZE
6	LGRE, RLGE, LRLGE, LGZE, SZLGE, LZLGE
7	LZHGE, LZE
8	Correlation

13 Patients did not have a hypoxic volume bigger than 1 ml. Taking SUVmax and the WHO grading into account, it was possible to improve the positive predictive value from 65.8 (if only expecting hypoxia) to 81.6%.

38 different heterogeneity parameters were calculated for the FDG PET and correlated with tumour hypoxia. Entropy showed the highest correlation with hypoxia (r = 0.52, p<0.001) of all TA parameters.

Entropy did only show a weak correlation to FMISO SUVmax (r = 0.24, p<0.001), tumour staging and WHO grading (below r<0.2) and no significant correlation to the HV (r = 0.35, p = 0.18). Logistic regression did not show significant results using TA parameter.

## Discussion

To our best knowledge, this study is the first trial to investigate voxel based analysis for predicting hypoxia by FDG PET and using texture analysis parameter to establish a better understanding of the relationship between hypoxia and increased glucose metabolism in tumours in a clinical setting. It was found that most head-and-neck tumours showed hypoxia which was moderately correlated to glycolysis both by SUVmax and voxel-wise. The concordance rate showed that 27% of the HMV was hypoxic. Vice versa the SUVmean in hypoxic tumour regions was twice as high as in the non-hypoxic regions.

FMISO PET is considered to be the standard non-invasive modality for evaluation of hypoxia [1–4,6]. Similar to our finding, a high rate of hypoxia in head-and-neck cancers has been demonstrated before [28]. FDG-PET revealed a good correlation with FMISO PET based on SUV values, voxel based and showed a positive concordance rate of 27% reflecting that the hypoxic fraction is smaller than the HMV.

SUVmean (FDG) ratio in hypoxic to non-hypoxic areas was around 2:1 in the current study. Theoretically, 16:1 might be expected by biochemistry in anoxic tissue. The discrepancy can be explained by 1) limited spatial resolution of PET showing a mixture of hypoxic and non-hypoxic cells, not anoxic (only hypoxic, FMISO starts accumulation below an oxygen level of 10 mmHg), 2) hypoxia may reduce metabolism and increase oxygen extraction fraction [29], 3) in hypoxic area, necrosis is developing and cell density is decreasing, FDG uptake may be decreased, 4) FDG uptake does not completely reflect the entire process of glycolysis, but rather reflects glucose transporters and the hexokinase activity only [30].

This agrees as well to the fact that oxygenation does not directly correlate with tumour cell proliferation as an increased amount of glucose is needed in hypoxic regions [31]. Our results emphasize that the information given by the FDG PET is not sufficient for localized treatment adaption of radiation therapy as dose painting [12].

Texture analysis, which is a group of methods to quantify the image heterogeneity, is an emerging subject in the field of medical imaging [20]. Hypoxic tumours may have different levels of metabolic heterogeneity than non-hypoxic tumours, because of elevated glucose consumption [32] (Warburg effect [33]) and decreased glucose uptake in necrotic areas (i.e., more variety of metabolic level). Therefore, we experimentally applied texture analysis to FDG PET image. Increased metabolic heterogeneity was found before in xenograft tumours [23] and this heterogeneity might lead to irregular benefits of drugs [34]. Intermediate strong and significant correlations were found between various texture parameters derived from the FDG PET images and hypoxia defined by SUVmax(FMISO) or HV. There were only low correlations between FDG TA parameters and WHO grading. Eight strongly correlating groups of TA parameter were found, similar group as found by Orlhac et al. 2014. FMISO texture analysis was not further investigated in the current study as it cannot replace FDG PET because of its inferior sensitivity in regard of tumour detection. Logistic regression was used to in combination with texture analysis to improve the detection of hypoxic tumours. Due to the established definition of hypoxia only 5 tumours were non-hypoxic. Therefore, the pre-predictive value is already 86.8% and logistic regression can improve this value to 92.1%. As there is a chance that very small hypoxic volumes may be related to image noise and/or have no impact we artificially divided the tumours small and big HV and were able to show that SUVmax and WHO grading had the highest impact to predict hypoxia.

Our results suggest that hypoxia and especially the hypoxic region within the tumour as visualized by FMISO PET cannot be predicted by the given TA parameters and FDG PET. Similarly, there were no correlation found between the necrosis detected in the MR image and hypoxia. One probable reason might be that the resolution of PET even with modern PET scanners using time-of-flight and low energy 18F-tracer is not high enough to visualize microscopic heterogeneity. A second reason is related to tumour size: as the head-and-neck tumours were relatively small, no macroscopic hypoxic/necrotic area was found in the included NCE-CT scan. It did not supply added value in ER+/HER2- breast cancer patients either [21]. Probably, TA might be more useful in larger tumours such as brain tumours and gynecological tumours which develop central necrotic lesions, or PET systems with higher (~10-fold) spatial resolution [7,35]. Third, in vitro experiments with glioma in rats showed that FMISO uptake can reflect the upregulation of GLUT1 transporters in hypoxic tumour cells but not the glycolysis [36]. As well it was shown in lung cancer patients that FDG and FMISO PET demonstrate different kinetics [37]. As the biology of cancers differ TA parameter can yield differently strong benefit on different cancer entities [38].

This study has some limitations. For best fusion of FDG and FMISO PET image sets, a mesh mask as used for radiation therapy would have been the best possible solution. For the patient convenience, this idea was abolished and standardised head positioning and automatic software fusion with minimal manual corrections was considered to be appropriate. The histology data did not give detailed information in regard of tumour hypoxia. Therefore, further analysis such as HIF-1 staining, MIB-1 staining was not possible. Due to software limitations, it was not possible to conduct TA of the MR data. The division in tumour with smaller and larger HV is artificial and needs further investigation.

## Conclusion

Moderate correlations were found between FDG PET and FMISO PET in the voxel-based analysis, with a two-fold higher uptake in FDG PET for hypoxic areas compared to non-hypoxic areas. However, the concordance rate showed that the hypoxic fraction is a smaller than the high FDG uptake volume. No TA parameter of the FDG PET correlated well with Hypoxia, TMR, HV measured in the FMISO PETs.

Our findings emphasize that there is no additional value in FDG PET to predict hypoxia compared to FMISO PET/CT and therefore it should not replace it in evaluating hypoxic areas in head and neck cancer. Translated into a clinical setting, hypoxia-targeted PET imaging remains necessary to assess hypoxia to investigating adopted treatment.

## Supporting information

S1 Dataset (DOCX)

S1 Table (DOCX)
