# Supplementary material for: Voxel based comparison and texture analysis of 18F-FDG and 18F-FMISO PET of patients with head-and-neck cancer
Source: PLoS One. 2019 Feb 28;14(2):e0213111. doi: 10.1371/journal.pone.0213111 (PMC6394953; doi:10.1371/journal.pone.0213111)
Supplement: S1 Dataset — (DOCX) [file pone.0213111.s001.docx]

**Supplemental dataset 1**

Detail of the texture analysis

**Histogram**

From 1-dimentional histogram, the following 5 features are computed.

$${SD}_{Hist}=\sqrt{\frac{1}{E-1}\sum_{i} \left( F\left( i \right)-\bar{F} \right)^{2}}$$

$${Skewness}_{Hist}=\frac{\frac{1}{E}\sum_{i} \left( F\left( i \right)-\bar{F} \right)^{3}}{\left( \sqrt{\frac{1}{E}\sum_{i} \left( F\left( i \right)-\bar{F} \right)^{2}} \right)^{3}}$$

$${Kurtosis}_{Hist}=\frac{\frac{1}{E}\sum_{i} \left( F\left( i \right)-\bar{F} \right)^{4}}{\left( \sqrt{\frac{1}{E}\sum_{i} \left( F\left( i \right)-\bar{F} \right)^{2}} \right)^{4}}$$

$${Energy}_{Hist}=\sum_{i} {p\left( i \right)}^{2}$$

$${Entropy}_{Hist}=-\sum_{i} p\left( i \right)\times\log\left( p\left( i \right) \right)$$

$E$ is the total number of the voxels in the VOI. $F(i)$ is the gray-level discrete intensity of *i*th voxel (*i* = 0, 1, 2, … , *E*-1). $\bar{F}$is the mean of $F(i)$. p(i) is the probability of *i*th bin. p(i) satisfies both $0\leq p\left( i \right)\leq1$ and $\sum_{i} p\left( i \right)=1$. In case that $p\left( i \right)=0$, $p\left( i \right)\times\log\left( p\left( i \right) \right)$is treated as 0 because $\lim_{x\to0} x\log x=0$.

**Gray-level co-occurrence matrix (GLCM)**

$C\left( i,j \right)$, the element of the *i*th row and *j*th column of GLCM, represents the probability of co-occurrence of M(x,y,z) = i and M(x+Δx, y+Δy, z+Δz) = j. M is the original voxel matrix. (Δx, Δy) can be either (-1,-1,-1), (0,-1,-1), (1,-1,-1), (-1,0,-1), (0,0,-1), (1,0,-1), (-1,1,-1), (0,1,-1), (1,1,-1), (-1,-1,0), (0,-1,0), (1,-1,0), or (-1,0,0), representing 13 different directions respectively.

$C\left( i,j \right)$ satisfies both $0\leq c\left( i,j \right)\leq1$ and $\sum_{i,j} c\left( i,j \right)=1$.$i$ and $j$ can be 0, 1, 2, … , n-1, where n is the number of gray levels. In the current study n was 64.

$${Homogeneity}_{GLCM}=\sum_{i} \sum_{j} \frac{C\left( i,j \right)}{1+\left| i-j \right|}$$

$${Energy}_{GLCM}=\sum_{i} \sum_{j} {C\left( i,j \right)}^{2}$$

$${Correlation}_{GLCM}=\sum_{i} \sum_{j} \frac{\left( i-\mu_{i} \right)\left( j-\mu_{j} \right)c\left( i,j \right)}{\sigma_{i}\sigma_{j}}$$

$${Contrast}_{GLCM}=\sum_{i} \sum_{j} \left( i-j \right)^{2}C\left( i,j \right)$$

$${Entropy}_{GLCM}=-\sum_{i} \sum_{j} C\left( i,j \right)\times\log\left( C\left( i,j \right) \right)$$

$${Dissimilarity}_{GLCM}=\sum_{i} \sum_{j} \left| i-j \right|C\left( i,j \right)$$

(µi,σi) or (µj,σj) represents the mean and standard deviation in *i*th row or *j*th column of C.

As mentioned above, each feature can be computed in 4-direction manners; thus, 4 different values are computed. In the current study, the average of 13 features were used as the representative values.

**Gray-level run-length matrix (GLRLM)**

$R\left( i,j \right)$, the element of the *i*th row and the *j*th column of the GLRLM, represents the number of homogeneous runs with gray-level *i* and length *j*. Similarly to GLCM, the direction (Δx, Δy, Δz) can be either (-1,-1,-1), (0,-1,-1), (1,-1,-1), (-1,0,-1), (0,0,-1), (1,0,-1), (-1,1,-1), (0,1,-1), (1,1,-1), (-1,-1,0), (0,-1,0), (1,-1,0), or (-1,0,0).

$$SRE=\frac{1}{\theta}\sum_{i} \sum_{j} R\left( i,j \right)\times\frac{1}{j^{2}}$$

$$LRE=\frac{1}{\theta}\sum_{i} \sum_{j} R\left( i,j \right)\times j^{2}$$

$$LGRE=\frac{1}{\theta}\sum_{i} \sum_{j} R\left( i,j \right)\times\frac{1}{i^{2}}$$

$$HGRE=\frac{1}{\theta}\sum_{i} \sum_{j} R\left( i,j \right)\times i^{2}$$

$$SRLGE=\frac{1}{\theta}\sum_{i} \sum_{j} R\left( i,j \right)\times\frac{1}{{i^{2}j}^{2}}$$

$$SRHGE=\frac{1}{\theta}\sum_{i} \sum_{j} R\left( i,j \right)\times\frac{i^{2}}{j^{2}}$$

$$LRLGE=\frac{1}{\theta}\sum_{i} \sum_{j} R\left( i,j \right)\times\frac{j^{2}}{i^{2}}$$

$$LRHGE=\frac{1}{\theta}\sum_{i} \sum_{j} R\left( i,j \right)\times i^{2}j^{2}$$

$$GLNUr=\frac{1}{\theta}\sum_{i} \left( \sum_{j} R\left( i,j \right) \right)^{2}$$

$$RLNU=\frac{1}{\theta}\sum_{j} \left( \sum_{i} R\left( i,j \right) \right)^{2}$$

$$RP=\frac{\theta}{\sum_{i} \sum_{j} \left( j\times R\left( i,j \right) \right)}$$

$\theta$ is the number of homogeneous runs within VOI, which is equal to $\sum_{i,j} R\left( i,j \right)$.

As mentioned above, each feature can be computed in 13-direction manners; thus, 13 different values are computed. The average of 13 features were used as the representative values.

**Gray-level zone-size matrix (GLZSM)**

GLZSM can be also called GLSZM, standing for gray-level size-zone matrix. $Z\left( i,j \right)$, the element of the *i*th row and the *j*th column of the GLZSM, represents the number of homogeneous zones with gray-level *i* and size *j*. Unlike GLCM and GLRLM, the direction is not considered. In the current study, 6 neighbors are considered to connected pixels.

$$SZE=\frac{1}{\theta}\sum_{i} \sum_{j} Z\left( i,j \right)\times\frac{1}{j^{2}}$$

$$LZE=\frac{1}{\theta}\sum_{i} \sum_{j} Z\left( i,j \right)\times j^{2}$$

$$LGZE=\frac{1}{\theta}\sum_{i} \sum_{j} Z\left( i,j \right)\times\frac{1}{i^{2}}$$

$$HGZE=\frac{1}{\theta}\sum_{i} \sum_{j} Z\left( i,j \right)\times i^{2}$$

$$SZLGE=\frac{1}{\theta}\sum_{i} \sum_{j} Z\left( i,j \right)\times\frac{1}{{i^{2}j}^{2}}$$

$$SZHGE=\frac{1}{\theta}\sum_{i} \sum_{j} Z\left( i,j \right)\times\frac{i^{2}}{j^{2}}$$

$$LZLGE=\frac{1}{\theta}\sum_{i} \sum_{j} Z\left( i,j \right)\times\frac{j^{2}}{i^{2}}$$

$$LZHGE=\frac{1}{\theta}\sum_{i} \sum_{j} Z\left( i,j \right)\times i^{2}j^{2}$$

$$GLNUz=\frac{1}{\theta}\sum_{i} \left( \sum_{j} Z\left( i,j \right) \right)^{2}$$

$$ZSNU=\frac{1}{\theta}\sum_{j} \left( \sum_{i} Z\left( i,j \right) \right)^{2}$$

$$ZP=\frac{\theta}{\sum_{i} \sum_{j} \left( j\times Z\left( i,j \right) \right)}$$

$\theta$ is number of homogeneous zones within VOI, which is equal to $\sum_{i,j} Z\left( i,j \right)$. ZSNU can be also called ZLNU, standing for zone-length non-uniformity.

**Neighborhood gray-level difference matrix (NGLDM)**

NGTDM is actually a column vector whose elements are s(i) (i=0, 1, 2, …, n-1, where n is the number of gray levels, for example 64). s(i) is the sum of $\left| i-\mu\right|$ about every voxel having gray-level discrete intensity *i*, where $\mu$ indicates the average of gray-level discrete intensity of the surrounding voxels excluding the central voxel. To avoid misunderstanding, let us explain using a sample image consisting of 5×5 pixels (left figure). Each value is assumed to be already discrete.


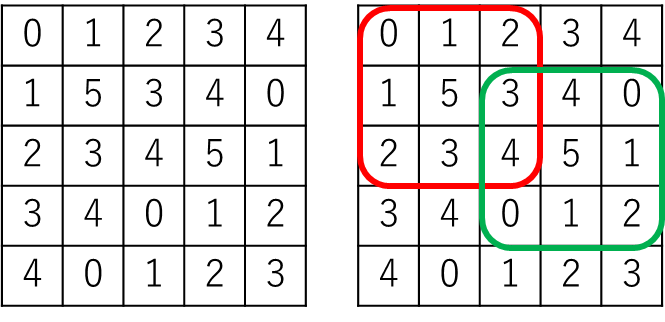


Consider computing s(5). Two different pixels of 5 are found in the image. The left figure shows red and green squares with center voxel being 5.

The average within red except the central voxel is (0+1+2+1+3+2+3+4)/8 = 2; |i – A|=|5-2|=3

The average within green except the central voxel is (3+4+0+4+1+0+1+2)/8 = 1.875; |i – A|=|5-1.875|=3.125

Thus, the sum of them is 3.0 + 3.125 = 6.125; s(5) = 6.125.

When computing the average of surrenders of edge pixels, there are 2 options. One choice is to compute the average of the voxels within the image. The other choice is to omit the computation of average for such voxels. In the current study, NGLDM was computed in the latter way. p(i) is the probability of occurrence of a voxel of gray-level i, which is same as histogram.

$$Coarseness=\left( \varepsilon+\sum_{i} p\left( i \right)s\left( i \right) \right)^{-1}$$

$${Contrast}_{NGLDM}=\frac{\left( \sum_{i} \sum_{j} p\left( i \right)p\left( j \right)\left( i-j \right)^{2} \right)\left( \sum_{i} s\left( i \right) \right)}{n\left( n-1 \right)E}$$

$${Busyness}_{NGLDM}=\frac{\sum_{i} p\left( i \right)s\left( i \right)}{\left| \sum_{i} \sum_{j} \left( ip\left( i \right)-jp\left( j \right) \right) \right|}$$

$\varepsilon={10}^{-20}$ prevents division-by-zero error.
