# Supplementary material for: Voxel based comparison and texture analysis of 18F-FDG and 18F-FMISO PET of patients with head-and-neck cancer
Source: PLoS One. 2019 Feb 28;14(2):e0213111. doi: 10.1371/journal.pone.0213111 (PMC6394953; doi:10.1371/journal.pone.0213111)
Supplement: S1 Table — (DOCX) [file pone.0213111.s002.docx]

**Supplemental table 1**

**Individual patient characteristics**

|  | **sex** | **age** | **origin** | **WHO grading** | **TNM** | **stage** |
| --- | --- | --- | --- | --- | --- | --- |
| 1 | m | 48 | Nasopharyngeal carcinoma | 3 | T1N1M0 | II B |
| 2 | f | 59 | Nasopharyngeal carcinoma | 2 | T4N1M0 | IV A |
| 3 | f | 45 | Nasopharyngeal carcinoma | 3 | T2bN2M0 | III |
| 4 | f | 66 | Nasopharyngeal carcinoma | 3 | T2aN0M0 | II A |
| 5 | m | 41 | Nasopharyngeal carcinoma | 2 | T3N1M0 | III |
| 6 | m | 77 | Nasopharyngeal carcinoma | 2 | T2bN2M0 | IV A |
| 7 | m | 56 | Oropharyngeal carcinoma | 2 | T2N2bM0 | III |
| 8 | m | 73 | Nasopharyngeal carcinoma | 3 | T4N1M1 | IV B |
| 9 | f | 62 | Nasopharyngeal carcinoma | unknown | T4N3bM1 | IV B |
| 10 | m | 52 | Nasopharyngeal carcinoma | 2 | T3N2M0 | III |
| 11 | m | 53 | Nasopharyngeal carcinoma | 2 | T1N3bM1 | IV C |
| 12 | m | 57 | Nasopharyngeal carcinoma | unknown | T3N2M0 | III |
| 13 | m | 66 | Nasopharyngeal carcinoma | 2 | T3N0M0 | III |
| 14 | f | 52 | Nasopharyngeal carcinoma | 2 | T1N1M0 | II B |
| 15 | m | 57 | Oropharyngeal carcinoma | unknown | T3N1M0 | III |
| 16 | m | 62 | Nasopharyngeal carcinoma | 2 | T3N3bM0 | IVB |
| 17 | m | 77 | Nasopharyngeal carcinoma | unknown | T1N0M0 | I |
| 18 | m | 63 | Nasopharyngeal carcinoma | 2 | T1N2M0 | III |
| 19 | m | 53 | Nasopharyngeal carcinoma | 2 | T3N2M0 | III |
| 20 | m | 44 | Nasopharyngeal carcinoma | 3 | T3N2M0 | III |
| 21 | f | 45 | Nasopharyngeal carcinoma | 2 | T4N1M0 | IV A |
| 22 | m | 67 | Nasopharyngeal carcinoma | 3 | T4N2M0 | IV A |
| 23 | m | 75 | Nasopharyngeal carcinoma | unknown | T4N2M0 | IVA |
| 24 | m | 62 | Nasopharyngeal carcinoma | 2 | T3N0M0 | III |
| 25 | m | 80 | Oropharyngeal carcinoma | unknown | T2N2bM0 | III |
| 26 | m | 61 | Nasopharyngeal carcinoma | 2 | T1N0M0 | I |
| 27 | m | 61 | Nasopharyngeal carcinoma | 2 | T4N0M0 | IV A |
| 28 | m | 52 | Nasopharyngeal carcinoma | 2 | T4N2M0 | IV A |
| 29 | m | 38 | Nasopharyngeal carcinoma | 3 | T2bN1M0 | IIB |
| 30 | m | 72 | Nasopharyngeal carcinoma | 3 | T1N0M0 | I |
| 31 | m | 60 | Nasopharyngeal carcinoma | 2 | T3N0M0 | III |
| 32 | f | 51 | Nasopharyngeal carcinoma | 1 | T3N0M0 | III |
| 33 | m | 62 | Nasopharyngeal carcinoma | 1 | T3N1M0 | III |
| 34 | m | 59 | Nasopharyngeal carcinoma | unknown | T2N1M0 | II |
| 35 | m | 57 | Nasopharyngeal carcinoma | 2 | T3N2M0 | III |
| 36 | m | 59 | Laryngeal cancer | 1 | T2N2bM0 | III |
| 37 | m | 71 | Oropharyngeal carcinoma | 2 | T3N2bM0 | III |
| 38 | m | 64 | Nasopharyngeal carcinoma | 2 | T3N1M0 | III |
